# Clinical findings of *Talaromyces marneffei* infection among patients with anti-interferon-γ immunodeficiency: a prospective cohort study

**DOI:** 10.1186/s12879-021-06255-9

**Published:** 2021-06-19

**Authors:** Zhao-Ming Chen, Zheng-Tu Li, Shao-Qiang Li, Wei-Jie Guan, Ye Qiu, Zi-Ying Lei, Yang-Qing Zhan, Hua Zhou, Sheng Lin, Xinni Wang, Zhun Li, Feng Yang, Wen Zeng, Ye Lin, Jing Liu, Jian-Quan Zhang, Feng Ye

**Affiliations:** 1 State Key Laboratory of Respiratory Disease, National Clinical Research Center for Respiratory Disease, Guangzhou Institute of Respiratory Health, The First Affiliated Hospital of Guangzhou Medical University, National Center for Respiratory Medicine, Guangzhou, 510120 China; 2 Department of Comprehensive Internal Medicine, The Affiliated Tumor Hospital of Guangxi Medical University, Nanning, 530021 Guangxi China; 3 Department of Infectious Diseases, The Third Affiliated Hospital of Sun Yat-Sen University, Guangzhou, 510630 China; 4 The First Affiliated Hospital, Zhejiang University School of Medicine, Hangzhou, 310003 Zhejiang China; 5 Department of Respiratory and Critical Care Medicine, Fujian Provincial Hospital, Shengli Clinical Medical College of Fujian Medical University, Fuzhou, 350001 Fujian China; 6 Department of Respiratory and Critical Medicine, The First Affiliated Hospital of Guangxi Medical University, Nanning, 530021 Guangxi China; 7 Department of Respiratory and Critical Medicine, The Eighth Affiliated Hospital, Sun Yat-Sen University, Shenzhen, 518000 Guangdong China

**Keywords:** Anti-IFN-γ autoantibodies, *Talaromyces marneffei*, Clinical features, Clinical outcome

## Abstract

**Background:**

*Talaromyces marneffei* (*T. marneffei)* infection has been associated with adult-onset immunodeficiency due to anti-IFN-γ autoantibodies. We aimed to investigate the clinical features of non-HIV-infected patients with *T. marneffei* infection in southern China.

**Methods:**

Between January 2018 and September 2020, we enrolled patients with *T. marneffei* infection who were HIV-negative (group TM, *n* = 42), including anti-IFN-γ autoantibody-positive (group TMP, *n* = 22) and anti-IFN-γ autoantibody-negative (group TMN, *n* = 20) patients and healthy controls (group HC, *n* = 40). Anti-IFN-γ autoantibodies were detected by ELISA. Clinical characteristics and clinical laboratory parameters were recorded.

**Results:**

Compared with anti-IFN-γ autoantibody-negative patients with *T. marneffei* infection, anti-IFN-γ autoantibody-positive patients did not have underlying respiratory disease; more frequently exhibited dissemination of systemic infections with severe pleural effusion; had higher WBC counts, C-reactive protein levels, erythrocyte sedimentation rates, and neutrophil and CD8^+^ T cell counts; had lower hemoglobin levels; and were more likely to have other intracellular pathogen infections. Most of these patients had poor outcomes despite standardized antimicrobial therapy.

**Conclusion:**

*T. marneffei*-infected patients with higher anti-IFN-γ autoantibody titers have more severe disease and complex clinical conditions.

## Introduction

Immune deficiency, which is caused by anti-interferon-γ autoantibodies (anti-IFN-γ autoAbs), is an adult immune deficiency syndrome that was first described among patients with mycobacterial infection [1]. Patients have high titers of serum anti-IFN-γ autoAbs, which can inhibit signal transducer and activator of transcription 1 (STAT1) phosphorylation and interleukin-12 production, resulting in severe dysfunction of the Th1 response [2–4] and increased risk of infection by multiple intracellular pathogens, including nontuberculous Mycobacterium (NTM), *Talaromyces marneffei* (*T. marneffei*), *Cryptococcus neoformans*, and other intracellular pathogens [1–12]. *Talaromycosis* is a severe deep mycosis that mainly involves organs rich in monocyte-macrophages (i.e., the lungs, liver, and lymph nodes) and can be categorized into localized and disseminated disease. Disseminated disease is characterized by severe systemic symptoms and a high mortality rate [13].

*T. marneffei* infection has long been associated with acquired immunodeficiency syndrome caused by human immunodeficiency virus (HIV) infection [14]. In some regions, such as southern China, *T. marneffei* infection has historically been the major opportunistic infection associated with acquired immunodeficiency syndrome [15]. However, *T. marneffei* infection has been increasingly reported among non-HIV-infected patients with impaired cell-mediated immunity [16], and immune deficiency syndrome caused by anti-IFN-γ autoAbs is an important risk factor [11, 12]. However, the impact of anti-IFN-γ autoAbs on *talaromycosis* progression has not been clearly described.

In this study, we compared the clinical features and laboratory findings between *T. marneffei*-infected patients with and without high titers of serum anti-IFN-γ autoAbs. We further evaluated the impact of anti-IFN-γ autoAbs on the dynamic disease course. Our findings provide more evidence for the diagnosis and treatment of non-HIV-infected patients with *T. marneffei* infection and will contribute to improved prognosis and a reduced mortality rate.

## Methods

### Participants

In this prospective, multicenter cohort study, patients with *T. marneffei* infection (group TM) were recruited between January 2018 and September 2020 from 7 academic centers [The First Affiliated Hospital of Guangzhou Medical University (Guangzhou); The Third Affiliated Hospital of Sun Yat-sen University (Guangzhou); The First Affiliated Hospital of Guangxi Medical University and The Affiliated Tumor Hospital of Guangxi Medical University (Nanning); The First Affiliated Hospital of Zhejiang University School of Medicine (Zhejiang); The Shengli Clinical Medical College of Fujian Medical University (Fuzhou) and The Eighth Affiliated Hospital of Sun Yat-Sen University (Shenzhen)]. The inclusion criteria were as follows: 1) No laboratory evidence of HIV infection; 2) Clinical and/or imaging manifestations of *T. marneffei* infection*;* 3) Microbiological or pathological findings identified from sputum, tracheal aspirate, bronchoalveolar lavage fluid (BALF), lung biopsy sample, pleural effusion, bone marrow smear, skin hydrolipidic film exudate or lymph node smear consistent with any of the following manifestations: a) visible detection of fungi (rounded or oval-shaped with an obvious transverse septum) by microscopy after Wright staining; b) isolation of pathogens from culture; or c) pathological examination revealing *T. marneffei* infection with pyogenic granulomatous changes, central necrosis, and massive monocyte-macrophage infiltration [17].

Healthy controls (group HC) with normal routine blood test findings and chest radiography were recruited from the health checkup center in The First Affiliated Hospital of Guangzhou Medical University.

Participants with anti-IFN-γ autoAb titers exceeding the 99th percentile of group HC were classified as anti-IFN-γ autoAb-positive. We further divided group TM into group TMP (anti-IFN-γ autoAb-positive) and group TMN (anti-IFN-γ autoAb-negative).

We excluded study participants who were less than 18 years of age; had autoimmune disease, cancer, or immunodeficiency; or had received immunosuppressive medications within the previous 3 months.

#### Clinical assessment

We measured the level of anti-IFN-γ autoAbs and recorded the clinical characteristics and laboratory findings upon admission. For healthy controls, we documented age, sex, and race or ethnicity only. Patients with *T. marneffei* infection (group TM) were followed up at weeks 1 and 2 and months 1, 3, 6, 9 and 12 after antifungal treatment; at follow-up, the levels of anti-IFN-γ autoAbs were detected, and the clinical conditions were recorded. The epidemiological and clinical characteristics, laboratory findings, treatment and outcome data were extracted into a standardized case report from the electronic medical records. The definition for each outcome was defined as follows: 1) ‘Cured’ were defined as patients’ symptoms and signs disappearing completely, with the lesions in the lung and other involved organs markedly or completely absorbed and the laboratory indexes related to infection normalized; 2) ‘Improved’ were defined as patients’ symptoms and signs improving, with no new signs or symptoms of lung or other organ infection appeared, the lesions in the lungs and other organs were absorbed or there was no obvious deterioration, and the laboratory indicators related to infection were improved; 3) ‘Ineffective’ were defined as patients’ symptoms and signs worsening, or new symptoms or signs of lung or other organ infection occurring, with progression or no improvement in the lesions in the lungs or other organs and worsening or lack of improvement in the laboratory parameters related to infection. 4) ‘Recurrence’ were defined as patients’ clinical symptoms improving or the pathogen detection being negative after effective treatment and then reappearing with signs of pathogen infection, pathogen detection being positive again, or both. 5) Death. If data were missing or clarification was needed, we then obtained the data by direct communication with the attending physicians and other health care providers. All case records were independently reviewed by two senior physicians (Z.M.C. and Y.L.).

#### Determination of anti-IFN-γ autoAb titers

Blood specimens were collected in 5 ml anticoagulant tubes. Serum was separated by centrifugation at 3000 rpm for 10 min and diluted 16-fold. The serum anti-IFN-γ autoAb titers were determined by using an enzyme-linked immunosorbant assay kit (USCN Life Science, Inc., Wuhan, China) based on the instructions of the manufacturer. The anti-IFN-γ autoAb titers were determined by comparing the optical density of the sample to the standard curve (detection range 3.12 ng/ml-200 ng/ml).

### Statistical analysis

Data were analyzed using IBM SPSS Statistics for Windows, version 23 (SPSS Inc., Chicago, Illinois), and a *P*-value < 0.05 indicated statistical significance. Categorical data are presented as numbers and percentages, and continuous data are presented as medians and ranges or interquartile ranges (IQRs). Comparison of continuous data was performed using the Kruskal-Wallis test or Mann-Whitney test; Fisher’s exact test was used to compare categorical variables. Comparison of the levels of anti-IFN-γ autoAbs between each study group was performed using Student’s t-test, the F-test and analysis of variance. Analyses of the associations between anti-IFN-γ autoAb levels and factors of interest were carried out using linear correlation with the Pearson correlation coefficient. Associations between various possible risk factors and this clinical syndrome were calculated as odds ratios (ORs) and 95% confidence intervals (CIs). Independent risk factors were identified by binary logistic regression to adjust for possible risk factors, defined as variables with *P < 0.05* in multivariable analysis.

## Results

**Fig. 1:**
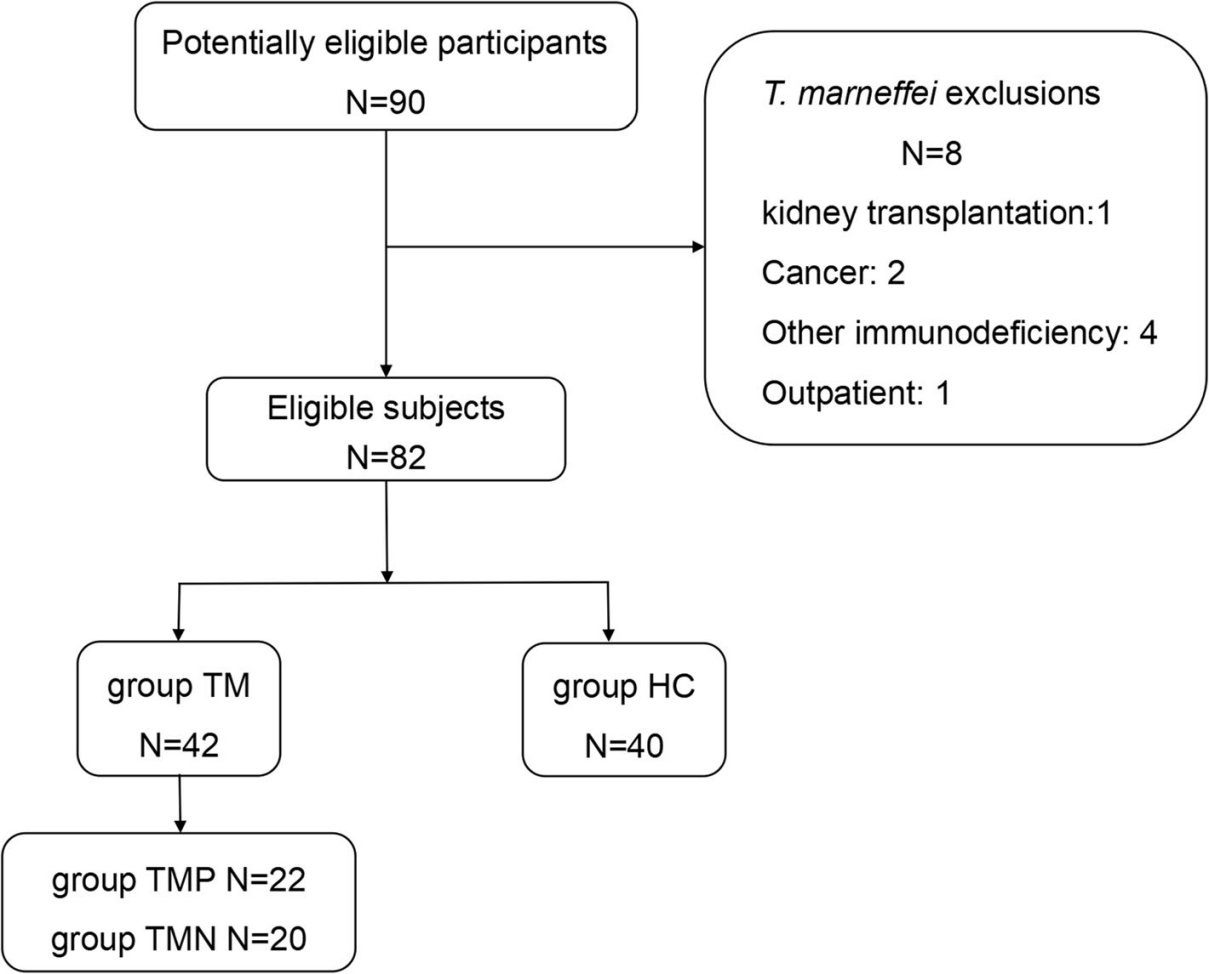
Flow chart of patient recruitment. Group TM = *Talaromyces marneffei.* Group TMP = anti-IFN-γ autoantibody-positive group. Group TNN = anti-IFN-γ autoantibody-negative group

Of the 82 eligible participants, 42 had disseminated *T. marneffei* infection (group TM) and 40 were healthy controls (group HC). The enrollment flow chart is shown in Fig. 1.

### Baseline characteristics and anti-IFN-γ autoAb titers

**Fig. 2:**
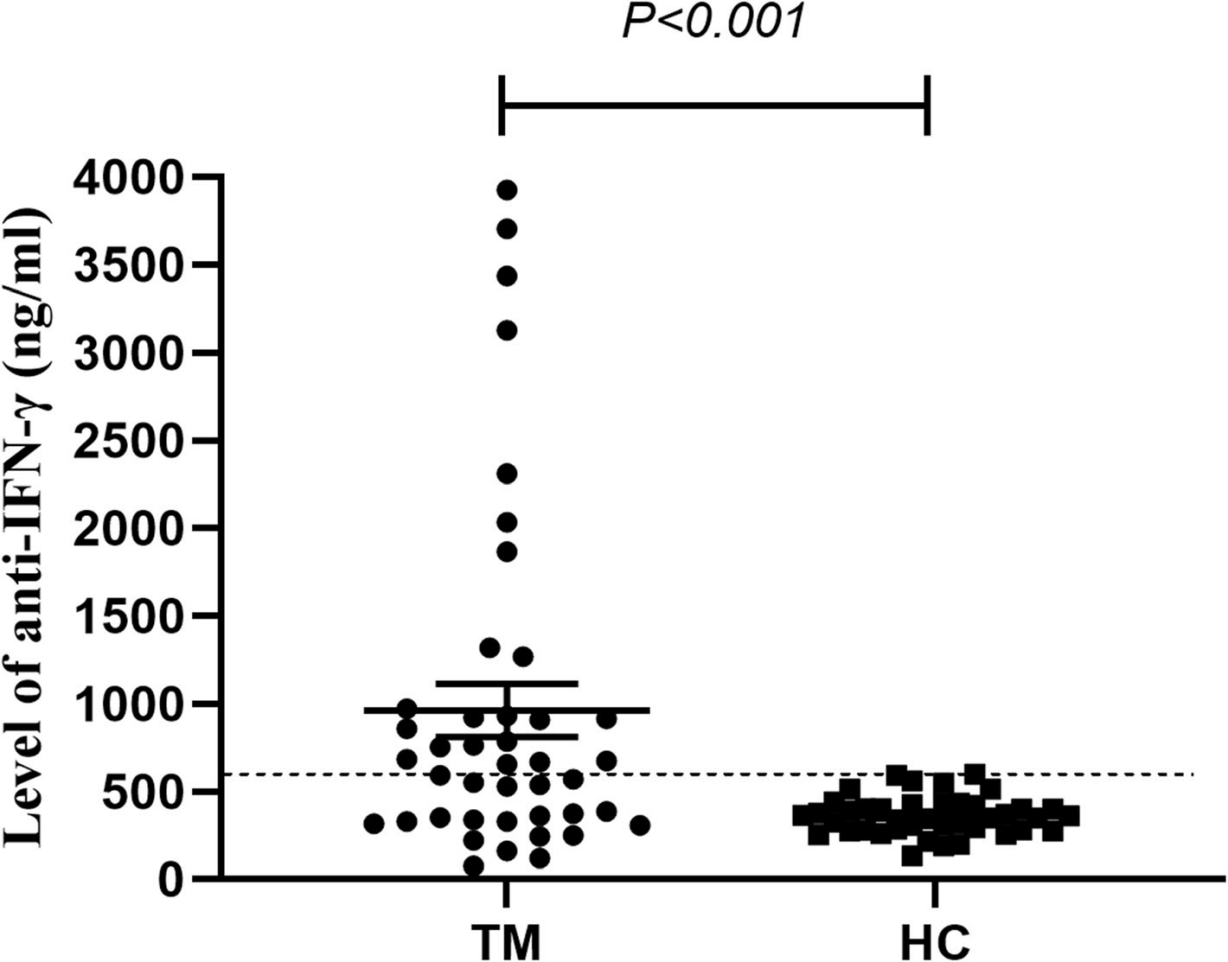
Levels of anti-IFN-γ autoAbs of atients with *T. marneffei* infection upon hospital admission and healthy controls. Each symbol represents an individual study participant. The dashed line indicates the estimated 99th percentile for the control group (group HC)

Sex distribution and age did not differ significantly between the two groups (Table 1). The anti-IFN-γ autoAb titers in group TM (median 661.33 ng/ml, range 334.88–941.29 ng/ml) were significantly higher than those in the healthy control group (median 353.97 ng/ml, range 277.40–422.38 ng/ml) (*P* < 0.001). Based on the 99th percentile of the anti-IFN-γ autoAb titers in group HC, the cutoff for anti-IFN-γ autoAb positivity was 594.49 ng/ml (Fig. 2). Twenty-two patients with *T. marneffei* infection were considered anti-IFN-γ autoAb-positive (group TMP) (Table 1).

**Table 1:** Baseline characteristics of the participants

Variable	Group TM (*N* = 42)	Group HC (*N* = 40)	*P v*alue
Age, years	53 (33, 59)	32 (27, 38)	0.646
Male sex, no. (%)	27 (64.3%)	18 (45.0%)	0.094
Anti-IFN-γ antibody-positive, no. (%)	22 (52.4%)	0	0.015
Anti-IFN-γ antibody titer (ng/ml)	661.33 (334.88, 941.29)	353.97 (277.40, 422.38)	< 0.001

### Comparison of clinical features between group TMP and group TMN

As shown in Table 2, no significant between-group differences were found when patients were stratified by age or sex. Significantly more patients with *T. marneffei* infection in group TMN than in group TMP had underlying respiratory disease (including chronic obstructive pulmonary disease, bronchiectasis and asthma) (*P* < 0.05). In addition to the lungs, the lymph nodes were the most common organs involved in both groups. Compared with patients in group TMN, patients in group TMP were more likely to have bone and skin involvement (*P* < 0.05). There were no significant between-group differences in the proportions of patients with fever, cough or sputum production, hemoptysis, dyspnea, chest pain, osteodynia or arthralgia, wasting, or moist rales. However, compared with patients in group TMN, patients in group TMP were more likely to develop pleural effusion (*P* < 0.05).

**Table 2:** Comparison of clinical characteristics between group TMP and group TMN during the first visit

Variable	Group TMP (*N* = 22)	Group TMN (*N* = 20)	*P value*
Age, years	52.0 (34.8, 58.0)	54.5 (29.0, 63.8)	0.734
Male sex, no. (%)	14 (63.6%)	13 (65.0%)	1.0
Anti-IFN-γ antibody titer (ng/ml)	926.31 (760.02, 2103.20)	332.40 (243.51,491.89)	<0.001
Time from symptom onset to diagnosis	149.5 (57.2, 272.3)	98.5 (22.3154.5)	0.107
**Coexisting respiratory disease, no. (%)**	3 (13.6%)	10 (50.0%)	0.019
Bronchiolitis	0	4 (20.0%)	0.043
COPD	2 (9.1%)	4 (20.0%)	0.400
COPD with bronchiectasis	0	2 (10.0%)	0.221
Asthma	1 (4.5%)	0	0.476
**Extrapulmonary organ involvement, no. (%)**	18 (81.8%)	11 (55.0%)	0.096
Skin	10 (45.5%)	3 (15.0%)	0.047
Lymph node	18 (81.8%)	11 (55.0%)	0.096
Liver	2 (9.1%)	1 (5.0%)	1.000
Spleen	2 (9.1%)	0	0.489
Bone	9 (40.9%)	1 (5.0%)	0.010
**Symptoms, no. (%)**			
Fever	12 (54.5%)	6 (30.0%)	0.131
Cough	17 (77.3%)	17 (85.0%)	0.700
Sputum production	14 (63.6%)	15 (75.0%)	0.514
Hemoptysis	4 (18.2%)	3 (15.0%)	1.0
Dyspnea	6 (27.3%)	3 (15%)	0.460
Chest pain	9 (40.9%)	6 (30.0%)	0.531
Osteodynia/Arthralgia	7 (31.8%)	3 (15.0%)	0.284
Wasting	13 (59.0%)	10 (50.0%)	0.757
Moist rales	6 (27.3%)	8 (40.0%)	0.515
Pleural effusion	13 (59.1%)	3 (15.0%)	0.005
Co-infection, no. (%)	12 (55.0%)	4 (20.0%)	0.029
**Outcome, no. (%)**^**a**^			
Cured	0	2 (10.0%)	–
Improved	2 (9.1%)	4 (20.0%)	–
Ineffective	6 (27.3%)	0	–
Recurrence	1 (4.5%)	0	–
Death	3 (13.6%)	0	–

^a^Data were available for 12 patients in group TMP and 6 patients in group TMN during the longitudinal follow-up

**Group TMP =** anti-IFN-γ autoantibody-positive group

**Group TMN =** anti-IFN-γ autoantibody-negative group

*COPD* Chronic obstructive pulmonary disease

*NTM* Nontuberculous Mycobacterium

The laboratory findings are shown in Table 3. Patients in group TMP had markedly higher leukocyte counts, neutrophil counts, eosinophil counts, erythrocyte sedimentation rates and C-reactive protein levels and lower hemoglobin levels than patients in group TMN (*P* < 0.05). Immunoglobulin levels were available for 32 patients. Patients in group TMP were more likely to have higher IgG antibody levels than those in group TMN. T cell counts were available for 25 patients, with median CD3^+^ T cell and CD8^+^ T cell counts of 1425.0 cells/μl and 631.0 cells/μl, respectively, in group TMP. These counts were significantly higher than those in group TMN. Modest between-group differences were identified when patients were stratified by the percentage of neutrophils and the platelet count.

**Table 3:** Comparison of laboratory findings between group TMP and group TMN during the first visit

Variable	Group TMP (*N* = 22)	Group TMN (*N* = 20)	*P value*
White cell count (*109 cells/L)	13.4 (9.3, 18.0)	7.7 (5.8, 12.0)	0.012
Absolute neutrophil count (*109 cells/L)	10.7 (6.9, 13.3)	5.1 (3.6, 11.4)	0.019
Neutrophil ratio (neut %)	0.76 (0.7, 0.9)	0.7 (0.6, 0.8)	0.120
Eosinophil count (*109 cells/L)	0.4 (0.1, 0.6)	0.1 (0.1, 0.3)	0.030
Hemoglobin (g/L)	89.7 (69.0,123.8)	111.0 (93.8129.8)	0.049
Platelet count (*109 cells/L)	310.5 (226.2485.7)	312.0 (189.0,408.0)	0.569
Erythrocyte sedimentation rate (mm/h)^a^	98.0 (72.0, 102.0)	52.0 (15.9, 88.0)	0.008
C-reactive protein (mg/dL)^b^	7.7 (3.3, 15.7)	72.5 (14.9, 137.2)	0.002
**Immunoglobulin**^c^			
Ig G (g/L)	28.7 (17.5, 34.0)	17.7 (13.7, 25.8)	0.026
Ig A (g/L)	1.1 (0.8,1.5)	1.2 (0.9,2.2)	0.400
Ig M (g/L)	2.6 (1.5, 2.8)	2.2 (1.5,2.9)	0.880
**T cell count**^d^			
CD3+ T cell count (cells/μl)	1425.0 (109.1, 1945.0)	830.5 (355.51302.3)	0.011
CD4+ T cell count (cells/μl)	685.0 (419.0, 965.5)	433.0 (111.3844.5)	0.247
CD8+ T cell count (cells/μl)	631.0 (499.0, 1132.0)	333.5 (151.0, 467.0)	<0.001

^a^Erythrocyte sedimentation rate data were missing for 8 patients (19%)

^b^C-reactive protein data were missing for 12 patients (28.5%)

^c^Immunoglobulin data were missing for 10 patients (23.8%)

^d^T cell count data were missing for 17 patients (40.5%)

**Group TMP =** anti-IFN-γ autoantibody-positive group

**Group TMN =** anti-IFN-γ autoantibody-negative group

**Fig. 3:**
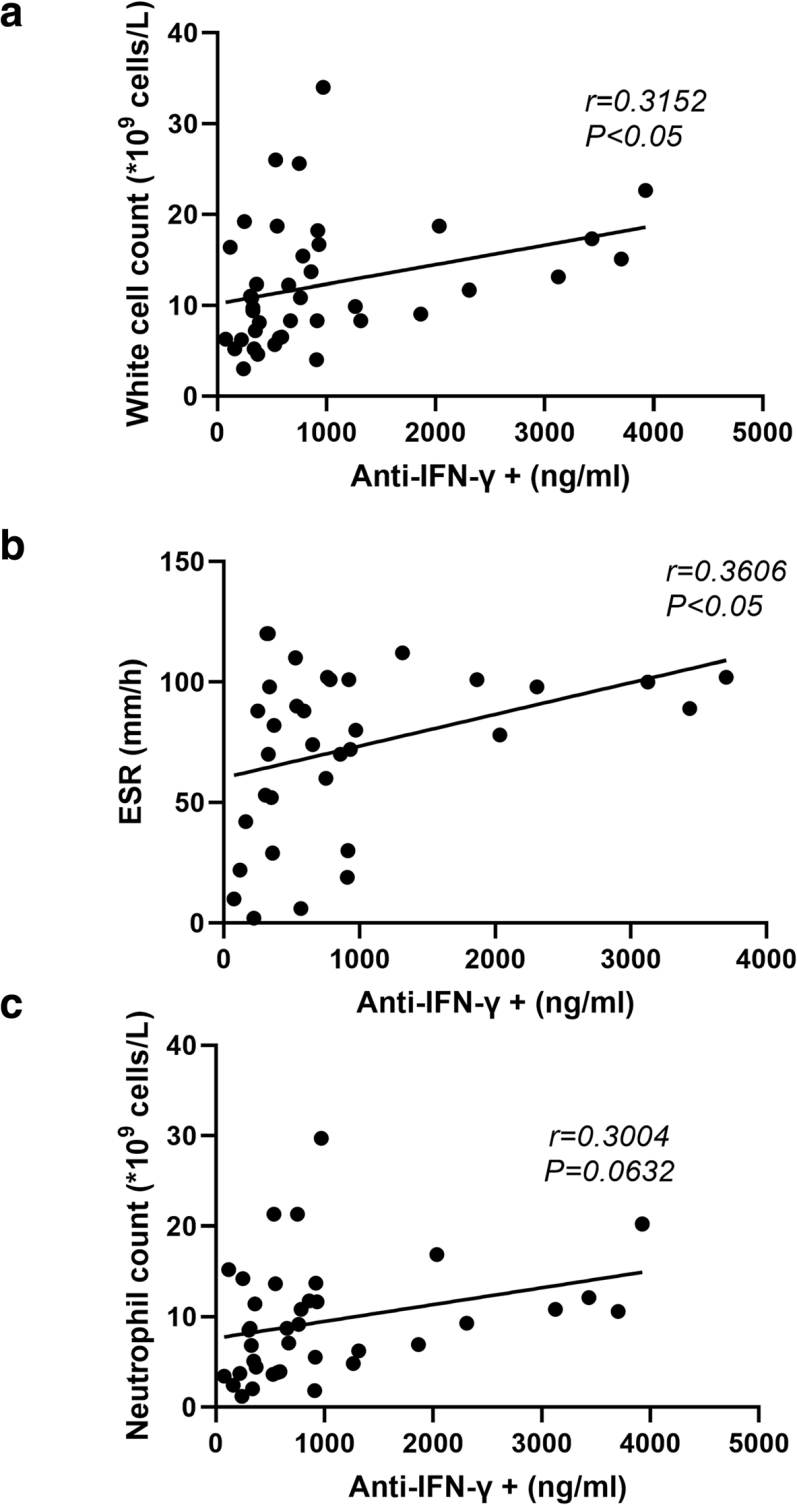
Pearson correlation analysis of serum anti-IFN-γ autoAbs and inflammatory markers among patients with *T. marneffei* infection. **a** Correlation between anti-IFN-γ autoAbs and white-cell count. **b** Correlation between anti-IFN-γ autoAbs and erythrocyte sedimentation rate. **c** Correlation between anti-IFN-γ autoAbs and absolute neutrophil count. ESR = Erythrocyte sedimentation rate

Moreover, the white blood cell count and erythrocyte sedimentation rate correlated significantly with the levels of anti-IFN-γ autoAbs in group TM (Fig. 3A-B). In addition, there was an apparent correlation between neutrophils and anti-IFN-γ autoAbs. (Fig. 3C). Univariate logistic regression analysis of patients in group TM showed that underlying respiratory disease (*P* = 0.031) and pleural effusion (*P* = 0.018) were independent factors associated with the appearance of anti-IFN-γ autoAb positivity (Table 4).

**Table 4:** Multivariate logistic regression analysis of factors associated with the appearance of anti-IFN-γ autoantibody positivity

Variable	Univariable OR (95% CI)	*P value*	Multivariable OR (95% CI)	*P value*
White-cell count (*10^9^ cells/L)	1.134 (1.007, 1.277)	0.038	1.035 (0.906, 1.183)	0.610
Hemoglobin (g/L)	0.973 (0.947, 1.001)	0.057	0.985 (0.948, 1.024)	0.454
Coexisting respiratory disease	**0.158 (0.035, 0.708)**	**0.016**	**0.094 (0.011, 0.809)**	**0.031**
Pleural effusion	**8.185 (1.839, 36.424)**	**0.006**	**11.162 (1.517, 82.164)**	**0.018**
Extrapulmonary organ involvement	3.682 (0.911, 14.876)	0.067	0.469 (0.046, 4.834)	0.525

*OR* Odds ratio, *CI* Confidence interval

### Clinical courses and outcomes of patients with T. marneffei infection

**Fig. 4:**
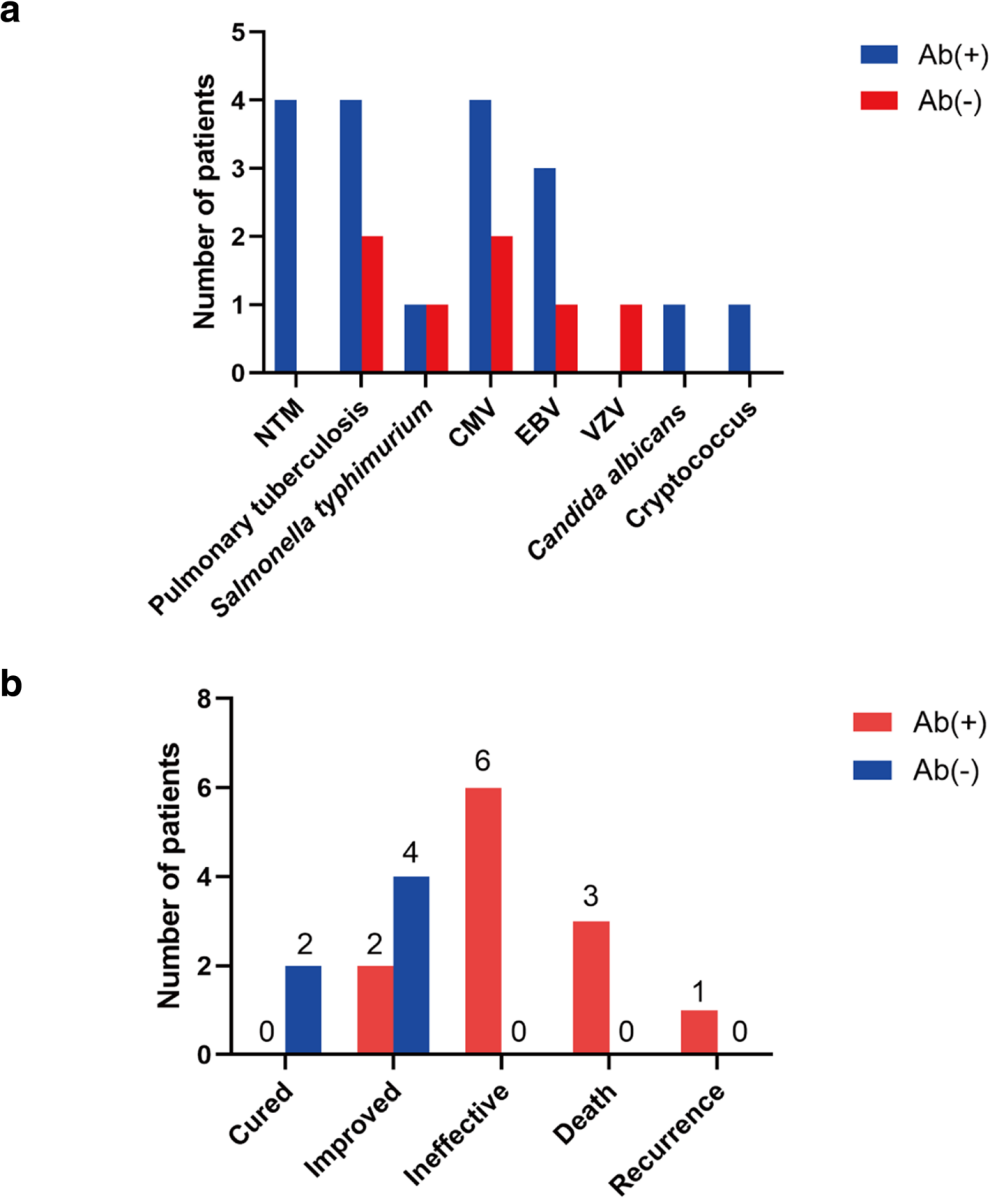
Clinical courses and outcomes of patients with *T. marneffei* infectio. **a** Coinfected pathogens among patients with *T. marneffei* infection. **b** Clinical outcome of patients with *T. marneffei* infection after 12 months of treatment*.* NTM = Nontuberculous Mycobacterium; CMV = Cytomegalovirus; VZV = Varicella zoster virus; EBV = Epstein-Barr virus

Of the 42 patients, 12 (55.0%) in group TMP and 4 (20.0%) in group TMN (*P* < 0.05, Table 2) were coinfected with other intracellular pathogens. Among patients in group TMP, 4 with disseminated NTM diseases (*n* = 4; 18.2%), 3 with active pulmonary *tuberculosis* and 1 with spinal *tuberculosis*, (*n* = 4; 18.2%), 4 with positive results of cytomegalovirus (CMV) DNA detection in BALF (*n* = 4; 18.2%), 3 with positive results of Epstein-Barr virus (EBV) DNA detection in BALF (*n* = 3; 13.6%), 1 with positive results of *Salmonella typhimurium* culture in sputum (*n* = 1; 4.5%), and 1 with positive results of *Candida albicans* culture in sputum (*n* = 1; 4.5%). Among patients in group TMN, 2 with active pulmonary *tuberculosis* (*n* = 2; 10.0%), 2 with positive results of cytomegalovirus (CMV) DNA detection in BALF (*n* = 2; 10.0%), 1 with positive results of Epstein-Barr virus (EBV) DNA detection in BALF (*n* = 1; 5.0%), 1 with positive results of *Salmonella typhimurium* culture in blood (*n* = 1; 4.5%), 1 with positive results of *Cryptococcus spp* culture in BALF (*n* = 1; 4.5%) and 1 with varicella zoster virus detected (VZV) DNA detection in blood (*n* = 1; 5.0%) (Fig. 4A).

In addition, data were available for 12 patients in group TMP and 6 patients in group TMN during the longitudinal follow-up. The treatment among patients included intravenous amphotericin B 0.6–1.0 mg/kg/day for 2 weeks, followed by oral itraconazole or voriconazole 400 mg/day for maintenance therapy. Despite long-term intensive treatment, patients in group TMP had a worse prognosis than patients in group TMN. In group TMP, 2 patients developed fatal disseminated infection and died within the first 30 postoperative days (premature mortality) because of multiple organ failure, 1 patient underwent 6 months of treatment and also complicated with secondary tuberculosis, and eventually died due to severe infections, 6 patients had persistent *T. marneffei* infection with a poor response after treatment. Furthermore, and one patient experienced clinical recurrence at 12 months. (Fig. 4B).

## Discussion

In this study, we confirmed that anti-IFN-γ autoAbs are an important risk factor for *T. marneffei* infection among non-HIV-infected patients. Most patients who were anti-IFN-γ autoAb-positive did not have any underlying respiratory disease and frequently had systemic dissemination with major pleural effusion. In addition, the leukocyte count and the levels of C-reactive protein and other inflammatory markers in these patients were significantly higher than those in anti-IFN-γ autoAb-negative patients. Despite the progression of *T. marneffei* infection, the anti-IFN-γ autoAb titer did not decrease after targeted treatment, and most patients had a poor outcome (such as death or recurrence of infection).

Anti-IFN-γ autoAbs have previously been shown to be an important risk factor for *T. marneffei* infections. Our data also supported this important finding. A large number of anti-IFN-γ autoAb-positive patients were found in group TM. This might be related to the high prevalence of anti-IFN-γ autoAb-associated HLA class II DRB1*16:02 and DQB1*05:02 alleles in the Asian population [18, 19]. Both Guangdong and Guangxi provinces are located in southern China, where *T. marneffei* are endemic due to the humid climate.

Since the 1990s, an increasing number of patients with *T. marneffei* infection have been reported among non-HIV-infected patients with impaired cell-mediated immunity. The comorbidities included primary adult-onset immunodeficiency due to anti-IFN-γ autoAbs and secondary immunocompromise, including that resulting from autoimmune disease or the use of immunosuppressive drugs such as novel anticancer targeted therapies and kinase inhibitors [16], but infections have been found even in patients with normal immunity [20]. The pathogenesis of anti-IFN-γ autoAb-positive patients was different from that of anti-IFN-γ autoAb-negative patients who had normal immunity and suffered from chronic lung diseases (such as COPD, bronchiectasis or asthma). Chronic lung diseases can lead to lung structural damage in various ways, resulting in impaired natural immune function and thereby changing the microenvironment that offers a niche for respiratory microorganisms. Furthermore, dysmicrobiosis might have increased the susceptibility of the hosts to *T. marneffei* infection [21, 22]. As a result, the lungs were the dominant organs involved, whereas anti-IFN-γ autoAb-positive patients were more likely to develop systemic dissemination of *T. marneffei* infection and pleural effusion [8]. Most patients with *T. marneffei* infection had pleural effusion characterized by yellowish exudates, with marked elevation of protein levels and nucleated cell counts [23]. Multiple organs might also be involved, especially the bone and skin, which might readily predispose patients to the development of Sweet syndrome [24].

Patients who tested positive for anti-IFN-γ autoAbs also had more significantly elevated inflammatory responses characterized by elevated leukocyte and neutrophil counts, erythrocyte sedimentation rates and C-reactive protein levels, markers indicating more exuberant infections in patients with anti-IFN-γ autoAbs. This is in accordance with previous studies such as by Angkasekwinai N et al. [25]. Neutrophils play an important role in the development of innate immunity. Neutrophils are the frontline barrier for eradicating the invasion of microbial pathogens and have powerful phagocytic capacity. In addition, neutrophils have been implicated in the production of the chemokine myeloperoxidase [26]. Neutrophil-derived IL-17A [27] also induces the release of IFN-γ, which promotes the antibacterial activity of macrophages [28]. We also observed a notable bone marrow response in this patient population, which was more prone to developing anemia [10] and leukocytosis [11]. A significant increase in CD8^+^ T cells was observed in anti-IFN-γ autoAb-positive patients. IFN-γ is indispensable for fighting infections because of its ability to regulate various protective functions and sustain the activity of both CD4^+^ and CD8^+^ T cells [29]. Conversely, IFN-γ produced by CD8^+^ T cells promotes the expression of major histocompatibility complex molecules on the surface of target cells to enhance antigen presentation and enhances the ability of macrophages and dendritic cells to phagocytose pathogens [30]. We speculate that the autoAbs might have affected the normal function of IFN-γ, leading to compensatory proliferation of these immune cells.

The patients were prospectively followed for up to 12 months, thus allowing us to estimate the correlation between the autoAb titer and disease progression. However, the anti-IFN-γ autoAb level did not correlate significantly with the clinical course, which was consistent with the findings from a previous study [31]. In addition, anti-IFN-γ autoAb-positive patients were more likely to be coinfected with other intracellular pathogens, especially NTM [32, 33]. Despite the initiation of standardized antimicrobial therapy, the patients’ conditions did not improve, and some deteriorated even further. There is no well-established standard therapy to reduce the titer of anti-IFN-γ autoAbs. However, one study reported a significant improvement in clinical symptoms after plasma exchange therapy [10]. Supplementation of IFN-γ recombinant protein [34], cyclophosphamide [35, 36] and B cell depletion with an anti-CD20 antibody [37, 38] have also been successfully used as adjuvant therapies in combination with antimicrobial therapy in a small number of patients. Prospective randomized clinical trials are needed to determine the therapeutic efficiency of these strategies.

Some limitations of our study need to be acknowledged. First, our sample size was relatively small, and therefore, selection bias might have affected the interpretation of our data. Second, for some patients, documentation of the laboratory findings during the longitudinal follow-up was incomplete, which hampered assessment of associations with the therapeutic response. Moreover, this was an observational cohort study with a limited duration of follow-up, and additional immunological experiments are needed to further explore the underlying mechanism.

## Conclusion

We outlined the clinical characteristics of *T. marneffei*-infected patients with immunodeficiency due to the presence of anti-IFN-γ autoAbs. These patients were more likely than anti-IFN-γ autoAb-negative patients to have systemic dissemination of infection and heightened inflammatory responses. More studies are needed to explore how to reduce the production of autoAbs, which might help to manage *T. marneffei infection* in patients with anti-IFN-γ autoAbs.

## Data Availability

The data-sets generated and/or analysed during the current study are not publicly available due to the presence of sensitive (confidential) participants’ information but are available from the corresponding author on reasonable request.

## Abbreviations

anti-IFN-γ autoAbs: Anti-interferon-γ autoantibodies
NTM: Nontuberculous Mycobacterium
*T. marneffei*: *Talaromyces marneffei*
HIV: Human immunodeficiency virus
IQRs: Interquartile ranges
COPD: Chronic obstructive pulmonary disease
Group TMP: Anti-IFN-γ autoantibody-positive group
Group TNN: Anti-IFN-γ autoantibody-negative group
ORs: Odds ratios
CIs: Confidence intervals
CMV: Cytomegalovirus
VZV: Varicella zoster virus
EBV: Epstein-Barr virus
